# Protein-truncating variants in *APBA1* in minors with severe, early-onset obesity: implications for genetic testing

**DOI:** 10.1186/s40348-026-00235-2

**Published:** 2026-04-22

**Authors:** Joanna Lerner, Abubakar Moawia, Stefanie Zorn, Simone Kloker, Margit Klehr-Martinelli, Kay Winner, Denisa Penfold, Thomas M. K. Völkl, Miriam Krausnick, Reiner Siebert, Martin Wabitsch

**Affiliations:** 1 Institute of Human Genetics, Ulm University Medical Centre, Ulm, Germany; 2 Centre for Rare Diseases, Ulm University Medical Centre, Ulm, Germany; 3 Division of Pediatric Endocrinology and Diabetes, Department of Pediatrics and Adolescent Medicine, Ulm University Medical Centre, Ulm, Germany; 4 German Center for Child and Adolescent Health (DZKJ), Partner Site Ulm, Ulm, Germany; 5 Division of Pediatric Endocrinology and Diabetology, Department of Pediatric and Adolescent Medicine, KJF Klinikum Josefinum, Augsburg, Germany; 6 Pediatric Endocrinology, Endokrinologikum München, Munich, Germany; 7 Institut Für Humangenetik, Universitätsklinikum Ulm, Albert-Einstein-Allee 11, Ulm, 89081 Germany; 8 Sektion Pädiatrische Endokrinologie Und Diabetologie, Universitätsklinikum Ulm, Klinik Für Kinder- Und Jugendmedizin, Eythstraße 24, Ulm, 89075 Germany

**Keywords:** *APBA1*, Hyperphagia, Obesity, Genetic Testing

## Abstract

**Background:**

Severe, early-onset obesity is a monogenic disorder in a subset of patients. Recently, rare protein-truncating variants (PTVs) in *BSN* and *APBA1* have been implicated as monogenic causes of obesity. Truncating variants in these two genes have been associated with adult-onset monogenic obesity. Here, we aimed to investigate the occurrence of rare PTVs in *APBA1* and *BSN* in minors with severe obesity manifesting in early childhood or adolescence.

**Methods:**

Previously obtained exome data of 209 children and adolescents (0–18 years) with severe obesity manifesting in early childhood or adolescence genetically tested at the Ulm University Medical Center between April 2022 and May 2024 were mined for rare PTVs in *APBA1* (NM_ 001163.4; NP_ NP_001154.2) and *BSN* (NM_003458.4; NP_003449.2). In all patients, monoallelic, and in the case of autosomal-recessive inheritance, biallelic likely pathogenic and pathogenic variants in well-known obesity-associated genes were excluded. Clinical re-evaluation was performed in two identified cases with rare potentially protein-truncating variants in *APBA1*.

**Results:**

The first patient (male, 15 years) presented with a BMI of 33.7 kg/m^2^ (BMI z-score: 2.7) after nine years of excessive weight gain despite life-style interventions. He had a body fat percentage of 49.5% and showed hyperphagia. The patient displayed impaired expressive language development, dyslexia and an IQ of 84. His mother also had obesity and dyslexia while his father and younger sister were unaffected. We identified the heterozygous, likely pathogenic variant *APBA1*:c.814C > T;p.(Gln272*) in the index patient and his mother. The second patient (male, 6 years) presented with a BMI of 30.0 kg/m^2^ (BMI z-score: 3.8), a body fat percentage of 43.6% and hyperphagia. The child had upslanting palpebral fissures and showed impaired expressive language development. Both parents of the boy were affected by severe obesity and underwent bariatric surgery. We identified the heterozygous variant of unknown significance *APBA1*:c.2442 + 3A > C predicted to impair proper splicing in the index patient and his mother.

**Conclusion:**

We conclude that rare potentially protein-truncating variants in *APBA1* are also found in some minors with early-onset and severe obesity. Therefore, those variants might not be restricted to adult-onset disease. We therefore propose to include *APBA1* in diagnostic genetic testing for monogenic obesity in minors and adults.

## Background

Identification of genes in which pathogenic genetic variants are possible causes for monogenic obesity, is not only important for gaining new insights into weight regulation pathways but particularly for the development of new therapeutic strategies and for instructing personalized treatment [19]. Currently, the leptin-melanocortin-pathway is considered to be the key pathway for the regulation of hunger, satiety and weight [19]. Monogenic obesity can be caused by alterations in genes which code for components of this pathway and in most cases disrupt its physiological function in the hypothalamus [19]. Among these well-known obesity-associated genes are *LEP*, *LEPR* and *MC4R* which code for Leptin, a hormone produced by adipocytes in response to sufficient caloric intake, its receptor and the Melanocortin receptor 4, which mediates satiety in the hypothalamus. Individuals with pathogenic disturbances of these pathways experience a massive weight gain starting in early childhood combined with often severe hyperphagia, a feeling of increased hunger and reduced satiety [12, 15]. Hyperphagia assessment in children with monogenic obesity using the Dykens’ Hyperphagia Questionnaire revealed values of 32.0 ± 9.3 and 31.4 ± 5.4 for children with biallelic *LEPR* and monoallelic *MC4R* variants respectively [29]. Patients without a known genetic cause of obesity reached notably lower values of 19.1 ± 7.9 [24]. BMI trajectories in early childhood showed a particularly rapid increase in children with biallelic *LEP*, *LEPR* and *MC4R* variants, surpassing the 99.9th BMI percentile by the age of six months, whereas children with monoallelic *MC4R* variants showed a less rapid and more continuous BMI increase [30].

In contrast to these early-onset forms of monogenic obesity, Zhao et al. recently identified genetic variants associated with adult-onset obesity in form of a monogenic trait in three adult populations of different ethnicity [27]. They propose that rare protein-truncating variants (PTVs) in *APBA1* and *BSN* may be among the few genetic determinants of predominantly adult-onset obesity based on their observation that these variants were not associated with increased childhood body weight. The variants’ effect on BMI is reported to be on par with that of heterozygous PTVs in *MC4R *[27]. The association between rare PTVs in *APBA1* and adult-onset obesity was replicated in the All of Us Research Program cohort, another adult cohort, further strengthening the relationship between this gene and weight regulation in adults [23].

In the case of *BSN*, rare PTVs have also been found in children and adolescents with severe obesity and not only in adults [27, 28]. In contrast, rare PTVs in *APBA1* have not yet been published in relation to pediatric manifestation of monogenic obesity. To explore whether PTVs in *APBA1* could also be related to childhood manifestation of monogenic obesity, we re-analyzed exome data of 209 children and adolescents (0–18 years of age) who were subjected to diagnostic genetic testing due to the suspicion of monogenic severe obesity manifesting in early childhood or adolescence between April 2022 and May 2024. In this cohort, we identified two rare heterozygous potential PTVs in *APBA1* in two minors with severe obesity and without likely pathogenic variants in other genes related to this phenotype, but no PTVs in *BSN*.

## Methods

### Study population

Pediatric patients with severe obesity manifesting in early childhood or adolescence subjected to diagnostic genetic testing at Ulm University Medical Center, Germany, were included in the re-evaluation of new obesity-associated genes for monogenic obesity between April 2022 to May 2024 (*n* = 209). The study group included 100 males and 109 females. The age range spanned from 0 to 18 years with a mean age of 9.7 ± 5.1 years (0–5 years: *n* = 57; 6–11 years: *n* = 69; 12–18 years: *n* = 83). All patients had undergone exome-based diagnostic panel-testing for well-known genetic forms of obesity beforehand. Written informed consent for genetic testing and participation in clinical research, including the use of genetic data for research purposes and publication of data, was obtained from the participants’ legal guardians in accordance with German law. The study on genetic causes of obesity in minors was conducted in accordance with the local legislation and institutional requirements and was approved by the Ethics Committee of Ulm University (352/21).

### Genetic testing

Whole-exome sequencing was performed using the TWIST™ Comprehensive Exome Kit (Twist Bioscience HQ, South San Francisco, USA) and the NextSeq High Output Kit v2.5 (300 cycles) (Illumina Inc., San Diego, USA). Short-read sequencing data processing, alignment to the hg19 reference genome (NCBI), variant calling, quality control and variant analysis were performed using varvis® software v2.1.0 (Limbus Medical Technologies GmbH, Rostock, Germany). Criteria used for variant calling included a minor allele frequency of < 1%, variants in non-coding regions 20 base pairs up- and downstream of coding regions and non-synonymous variants. All patients received a panel that included the following 38 genes related to monogenic or syndromic obesity: *ALMS1, ARL6, ASIP, BBS1, BBS10, BBS12, BBS2, BBS4, BBS5, BBS7, BBS9, CEP19, CEP290, CPE, GNAS, HTR2C, INPP5E, KIDINS220, KSR2, LEP, LEPR, MC4R, MKKS, MKS1, MRAP2, MYTIL, NTRK2, PCSKI, PGM2L1, PHF6, PHIP, POMC, SDCCAG8, SH2B1, SIM1, TTC8, TUB, VPS13B.*

In both patients with *APBA1* variants and the first patient’s first-degree relatives, a panel of 105 genes associated with monogenic or syndromic obesity was performed in order to search for alternative genetic causes. This panel included the following genes: *ADCY3, AFF4, ALMS1, ARL6, ASIP, BBIP1, BBS1, BBS10, BBS12, BBS2, BBS4, BBS5, BBS7, BBS9, BDNF, CCDC28B, CEP19, CEP290, CFAP418, CLOCK, CPE, CREBBP, CRHR2, CUL4B, DNMT3A, DYRK1B, EP300, FLOT1, G6PC1, GNAS, HTR2C, IFT172, IFT27, IFT74, INPP5E, IRS1, IRS2, IRS4, ISL1, KIDINS220, KSR2, LEP, LEPR, LZTFL1, MC3R, MC4R, MCHR1, MECP2, MKKS, MKRN3, MKS1, MRAP2, MYT1L, NCOA1, NDN, NPHP1, NR0B2, NRP1, NRP2, NTRK2, PAX6, PCK1, PCNT, PCSK1, PGM2L1, PHF6, PHIP, PLXNA1, PLXNA2, PLXNA3, PLXNA4, POMC, PPARG, PRKAR1A, PROK2, PTEN, RAB23, RAI1, RPGRIP1L, RPS6KA3, SCAPER, SCLT1, SDCCAG8, SEMA3A, SEMA3B, SEMA3C, SEMA3D, SEMA3E, SEMA3F, SEMA3G, SH2B1, SIM1, SNRPD2, SNRPN, SPG11, TBX3, THRB, TMEM67, TRIM32, TRPC5, TTC8, TUB, UCP3, VPS13B, WDPCP.* The coverage was a minimum of 20 reads for all patients and analyzed genes with the exception of *PHIP*. Exon 3 of the PHIP gene did not meet this criterium and was therefore excluded from the analysis. Detected variants were classified according to the guidelines of the American College of Medical Genetics and Genomics (ACMG); [22]. The Integrative Genomics Viewer (IGV) was used for the manual examination and visualization of the *APBA1* variants [14]. The clinical databases ClinVar, LOVD and HGMD professional and the current literature via the database Medline (PubMed) were searched for the identified variants. For splice prediction, we submitted the sequence in the region affected by the variant to Splice AI. https://spliceailookup.broadinstitute.org/) [13].

For the second *APBA1* variant carriers’ mother targeted sequencing was performed to determine carrier status. Genomic DNA was extracted from the obtained blood sample. Exon 12 of the *APBA1* gene was amplified by polymerase chain reaction (PCR) and subsequently sequenced in both the 5′ and 3′ directions. The resulting sequence data, generated using a capillary sequencer, were analyzed in comparison with the reference sequence (ENST00000265381.4; hg19) as well as with the sequence of the index patient.

In order to visualize the position of the two detected variants within the gene, a lollipop plot of *APBA1* (NM_001163 | ENST00000265381) was created via cBio Cancer Genomics Portal [4]. Protein expression was determined by using The Human Protein Atlas [26].

### Phenotypic characterization

Body height was measured via stadiometer (Busse Design, Ulm, Germany) to the nearest of 0.1 cm and body weight was assessed via calibrated scale to 0.1 kg (Seca, Hamburg, Germany). Body mass index (BMI, kg/m^2^) was calculated and standardized as BMI Standard Deviation Score (SDS, z-score) according to the German growth reference data [16, 17]. Obesity was defined as BMI ≥ 97. age- and sex-specific reference percentile and severe obesity as BMI > 99.5. age- and sex-specific reference percentile. The percentiles used correspond to the male reference percentiles according to [16] and [17]. Family history was assessed, including weight and height data from parents, and siblings if available, comorbidities, and pedigree. Early-childhood data on weight and height were collected from the national early childhood screening examinations from birth to five years of age. Hyperphagia was assessed at the first outpatient visit using the hyperphagia questionnaire from Dykens et al. [9]. Furthermore, an ad libitum test meal in a buffet style was performed and care givers were asked to fill out a food protocol two days prior to, on the day of and two days after the ad libitum test meal. The test meal was conducted in the morning after a minimum of eight hours overnight fast. The items were covertly weighed before and after the meal and total energy intake and energy intake per kilogram lean mass were calculated. Body composition and body fat mass (%) were assessed using Dual-energy X-ray absorptiometry (Hologic Inc., Marlborough, United States). Abdominal sonography was performed by a DEGUM II certified examiner using a GE LOGIQ-E10 ultrasound system (GE Healthcare GmbH, Düsseldorf, Germany). Standardized laboratory testing included the parameters HbA1c, fasting insulin, cholesterol, triglycerides, thyroid-stimulating hormone (TSH), total leptin serum concentration and bioactive leptin concentration. Leptin z-score and reference values for total leptin serum concentration were calculated by using the online tool provided by Brandt-Heunemann et al. [3].

## Results

In order to examine the presence of rare heterozygous potentially protein-truncating variants in *APBA1* and *BSN* in minors, we mined existing diagnostic exome data of 209 children and adolescents between the ages of 0 to 18 years (mean: 9.7 ± 5.1 years; 100 male, 109 female) who presented with severe obesity manifesting in early childhood or adolescence between April 2022 and May 2024. Only minors without likely pathogenic or pathogenic variants in at least 38 obesity associated genes were included. In this cohort, we detected no PTVs in *BSN*. However, we identified two cases with rare heterozygous potentially protein-truncating variants in *APBA1* and no other known monogenic causes of obesity.

### Case 1

**Fig. 1:**
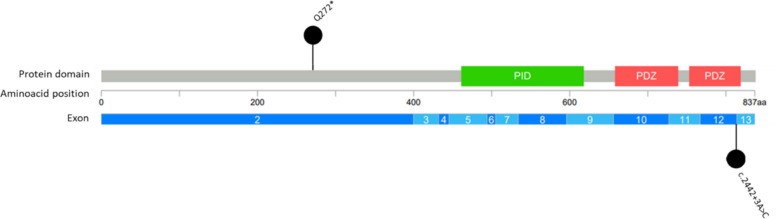
Lollipop plot of *APBA1* (NM_001163 | ENST00000265381) as created via cBio Cancer Genomics Portal [4]. The stop-gain variant identified in the first case (Q272*) is depicted according to its amino acid position. The splice-donor variant identified in the second case (c.2442 + 3 A > C) is displayed according to its position between the exons 12 and 13 of *APBA1.* All annotations, coordinates, and variant descriptions were based on GRCh37/hg19 assembly (NM_ 001163.4; NP_001154.2). PID: Phosphotyrosine interaction domain (PTB/PID) (461–618), PDZ: PDZ domain (658–739 and 753–819)

The first variant, *APBA1*:c.814C > T;p.(Gln272*) (NM_ 001163.4; NP_001154.2) was observed in a 15-year old boy (Fig. 1). The likely pathogenic heterozygous variant (ACMG criteria: PVS1, PM2), identified in the boy and his mother, was absent from gnomAD (v4.1.0) and clinical databases (ClinVar, HGMD professional and LOVD). The single nucleotide exchange leads to the introduction of an early stop-codon which in turn is predicted to lead to a nonsense-mediated mRNA decay and thus a loss of function. In the absence of any other causative variants in obesity-associated genes, we assume that this *APBA1* variant is the main cause for the described phenotype in our patient.

**Fig. 2:**
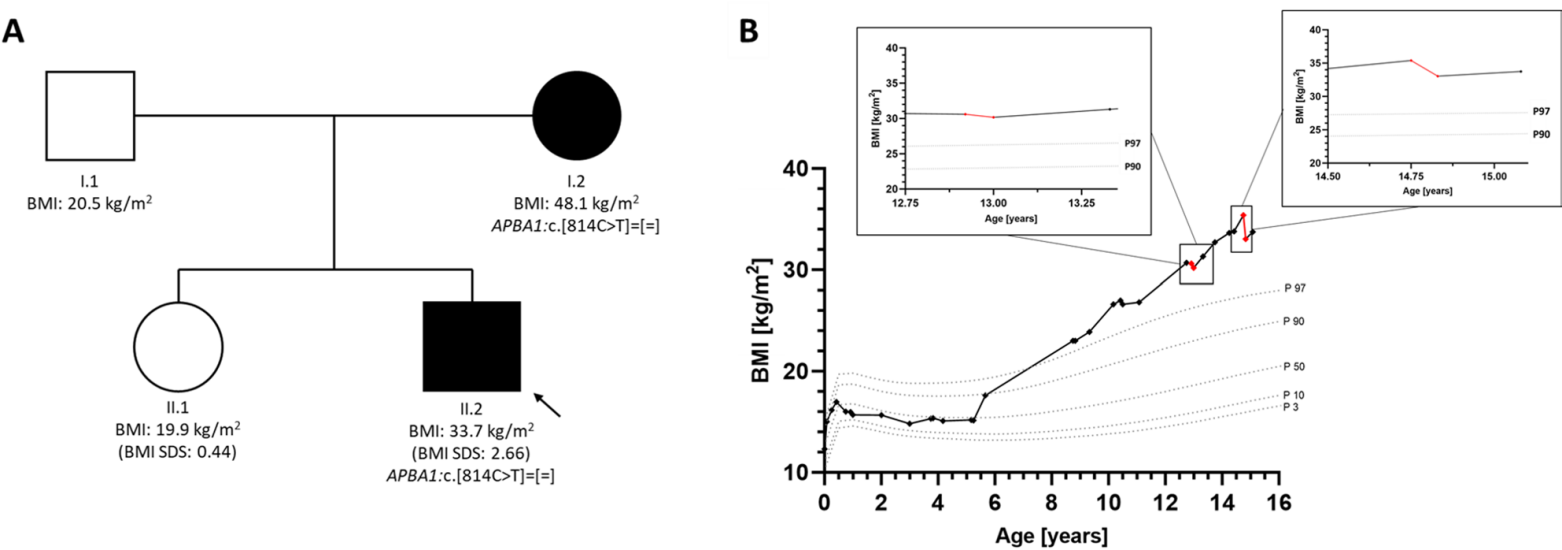
**A** Pedigree of the index patient’s first-degree relatives including the BMI-z-score at the time of assessment. Squares represent males, circles indicate females. Filled out symbols represent persons with severe obesity. Variant is noted according to the HGVS nomenclature. The black arrow indicates the index patient. **B** BMI trajectory of the index patient of the first family from early childhood to present. The effect of in-patient lifestyle intervention programs on BMI is indicated by the red segments in the graph, with each area magnified in a separate graph. The duration of each lifestyle intervention program was four weeks, and each magnification presents a time frame of seven months. All percentiles correspond to the male reference percentiles according to [16] and [17]

The boy presented after a nine-year history of excessive weight gain despite several life-style interventions including two in-patient weight loss programs with a duration of four weeks each (Fig. 2B). He presented with a BMI of 33.7 kg/m^2^ (BMI z-score: 2.7), a body fat percentage of 49.5% and signs of hyperphagia as indicated by the Dykens’ Hyperphagia Questionnaire with a total score of 27/55 points. In an ad libitum test meal, the boy consumed a total of 343.5 kcal (8.2 kcal per kilogram lean mass). The food protocol two days before, on the day of and two days after the test meal, showed a mean daily caloric intake of 1709 kcal (± 382.3 kcal) and no compensatory eating behavior. Laboratory findings showed elevated cholesterol and thyroid-stimulating hormone levels at the time of assessment (Tab. 1). Serum leptin levels were well above the 50th BMI- and pubertal stage-matched reference percentile (Leptin-z-score: 1.4, Tab. 1). Abdominal ultrasound revealed no organ malformations and no signs of liver steatosis. In the absence of dysmorphic features, the patient showed impaired expressive language development, dyslexia and an IQ of 84. He received extensive speech therapy and requires specialized schooling. Motor developmental milestones were reached within expected time frames. Although the boy was reported to have concentration problems, low frustration tolerance and was easily distracted, a standardized assessment for attention deficit hyperactivity disorder (ADHD) at the age of 11 years did not confirm this diagnosis. Further neurological examination including an EEG did not reveal any pathologies. The boy was born at term as the first child of non-consanguineous German parents. Whole-exome based panel testing was performed on the parents and the younger sister. He inherited the *APBA1* variant from his mother who shared both obesity (BMI: 48.1 kg/m^2^) and dyslexia with her son while his father and younger sister were unaffected (Fig. 2A). Interestingly, the mother reports being merely slightly overweight as a child and experiencing extreme weight gain only after her first pregnancy at age 27.

**Table 1:** Selected laboratory findings

	**Case 1**	**Case 2**
Age [years; months]	15;1	6;2
HbA1c [%]	5.4	5.4
Insulin, fasting [mU/l]	17.2	71.1
Cholesterol, total [mmol/l]	5.8	4.3
Triglycerides [mmol/l]	2.1	1.0
TSH^a^ [mlU/l]	5.9	5.1
Leptin, total [µg/l] (Leptin, z-score)	59.1 (1.4)	37.2 (−0.4)

The reference range for HbA1c was 4.0–6.0%

The reference range for fasting insulin was 2.6–24.9 mU/l

The reference range for total cholesterol was < 5.0 mmol/l

The reference range for triglycerides was < 1.7 mmol/l

The age-matched reference range for thyroid-stimulating hormone was 0.5–4.2 mlU/l for case 1 and 0.6–4.7 mlU/l for case 2

The age-, sex-, BMI- and Tanner stage matched reference percentiles for total leptin were 10.6 µg/l (3^rdt^ percentile), 32.9 µg/l (50th percentile) and 74.0 µg/l (97th percentile) for case 1 and 15.8 µg/l (3rd percentile), 44.5 µg/l (50th percentile), and 104.5 µg/l (97th percentile) for case 2. All reference values and z-scores were calculated using the leptin calculator [3]

^a^TSH: Thyroid-stimulating hormone

### Case 2

The second variant, *APBA1*:c.2442 + 3 A > C (NM_ 001163.4; NP_001154.2) was identified in a six-year-old boy with early-onset obesity (Fig. 1). In this boy and his mother, we identified the heterozygous *APBA1* variant of unknown significance (ACMG criteria: PM2, PP3), which was absent from gnomAD (v4.1.0) and clinical databases (ClinVar, HGMD professional and LOVD) and SpliceAI predicted the variant to possibly affect splicing due to an alteration of a splice donor site (delta score: 0.5) [13]. Protein truncation may be the result of such an alteration. Expression of *APBA1* in blood is very limited and it is mainly expressed in the central nervous system [26]. Therefore, we did not perform RNA-sequencing and could not confirm altered splicing. As in the first case, we have not found any other causative variants in obesity-associated genes matching the child’s phenotype. We assume that the *APBA1* variant could be the cause of the early-onset obesity.

**Fig. 3:**
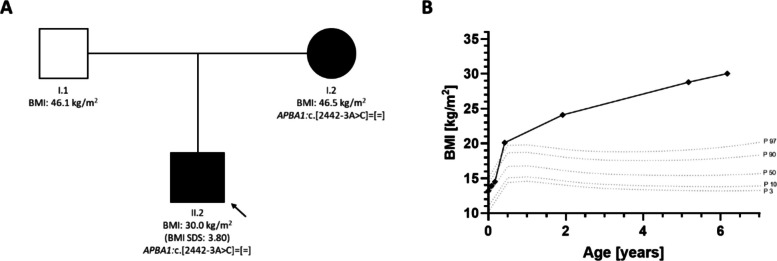
**A** Pedigree of the index patient’s first-degree relatives including the BMI-z-score at the time of assessment. Squares represent males, circles indicate females. Filled out symbols represent persons with obesity. Variant is noted according to the HGVS nomenclature. The black arrow indicates the index patient. **B** BMI trajectory of the index patient of the second family from early childhood to present. All percentiles correspond to the male reference percentiles according to [16] and [17]

Rapid weight gain started directly after birth (Fig. 3B). At the time of assessment, he presented with a BMI of 30.0 kg/m^2^ (BMI z-score: 3.8), a body fat percentage of 43.6% and clear signs of hyperphagia as indicated by the Dykens’ Hyperphagia Questionnaire with a total score of 32/55 points. In the ad libitum test meal, the boy consumed a total of 1046.1 kcal (35.5 kcal per kilogram lean mass). The food protocol two days before and on the day of the test meal, showed a mean daily caloric intake of 1917.3 kcal (± 622.7 kcal). Compensatory eating behavior could not be evaluated as there was no food protocol available for the two days after the test meal. Laboratory findings showed elevated fasting insulin and thyroid-stimulating hormone levels at the time of assessment (Tab. 1). Serum leptin levels were below the 50th BMI- and pubertal stage-matched reference percentile (Leptin-z-score: −0.4, Tab. 1). Abdominal ultrasound revealed no organ malformations and only mild signs of liver steatosis. The boy had upslanting palpebral fissures but no other dysmorphic features. He showed pronounced impaired expressive language development and was not able to form full sentences at the age of six years. Motor developmental milestones were slightly delayed, possibly due to his weight and body circumference. He received extensive speech and movement therapy from the age of five years. He was born at term as the first child of non-consanguineous parents who were both affected by severe obesity and both underwent bariatric surgery (Fig. 3A). His father (BMI_max_: 46.7 kg/m^2^) was neither available for clinical nor for genetic assessment. Any information about the father was obtained in conversation with the child’s mother. The boy’s mother had a maximum weight of 125 kg (BMI_max_: 46.5 kg/m^2^) and underwent bariatric surgery four month prior to her son’s first outpatient visit. She carried the same *APBA1* variant and reported hyperphagia. Similar to the reports of the first patient’s mother, the second patient’s mother also describes being slightly overweight as a child and experiencing excessive weight gain in her late teens after the pregnancy with her son.

## Discussion

In a cohort of 209 minors with severe obesity manifesting in early childhood or adolescence, we detected two cases with rare heterozygous PTVs in *APBA1* and no other known monogenic causes of obesity. Both minors inherited the variants from their affected mothers. In both cases, obesity was accompanied by hyperphagia and impaired expressive speech development. Speech impairment was reported to be developmental as opposed to regressive.

A recent association study was able to replicate the relationship between rare PTVs in *APBA1* and adult obesity first reported by Zhao et al. in the adult All of Us Research Program cohort, further strengthening the association between this gene and weight regulation in adults [23]. The observation that both mothers in our cases experienced excessive weight gain in adulthood also supports this association to adult-onset obesity. Zhao et al. propose that adult-onset obesity in carriers of rare PTVs in the *APBA1* and *BSN* genes is caused by neurodegeneration and vesicular transport dysfunction related to aging [27]. However, aging and neurodegeneration of appetite-regulating neurons as possible underlying mechanisms of obesity might be less likely to cause the early disease manifestation in the cases reported here, though it cannot be excluded particularly considering the neurological phenotype. Theoretically, APBA1 protein function could influence hunger, food intake and body weight at any age through its involvement with extracellular NMDA-receptors. APBA1 is a member of a transmembrane protein family that is essential for the exocytosis of NMDA-receptors into the extracellular department [21]. Studies in rats have demonstrated that chronic inactivation of extracellular NMDA-receptors disrupts the reduction of food intake after exposure to a high fat diet [5]. Consequently, those rats consumed up to twice the amount of food compared to control animals with intact extracellular NMDA-receptors 5. Therefore, it could be possible that APBA1 deficiency reduces the number of extracellular NMDA-receptors which in turn disturbs the regulation of caloric intake.

In our patients, the dysregulation of caloric intake manifests as hyperphagia. Additionally, both affected mothers report feelings of increased hunger, themselves. Hyperphagia assessment using Dykens’ Hyperphagia Questionnaire showed a total score of 27/55 points in the first and 32/55 in the second case. These scores fall within the age-matched reference for minors with Prader-Willi-Syndrome [9]. Similarly, these scores are comparable to that of individuals with Bardet-Biedl-Syndrome with 27.6 ± 9.0 23, biallelic *LEPR* (32.0 ± 9.3) or monoallelic *MC4R* (31.4 ± 5.4) mutations [29]. Notably, our patients’ values are above the range of patients without a known genetic cause of obesity (19.1 ± 7.9) [24].

Caloric intake in our patients was further evaluated by conducting an ad libitum test meal. Interestingly, the two boys differed greatly in their energy intake during this assessment despite both achieving high values in the Dykens Hyperphagia Questionnaire. The 15-year-old boy consumed 8.18 kcal per kilogram of lean mass while the six-year-old boy consumed 35.46 kcal per kilogram of lean mass. The latter value matches the caloric intake of patients with mono- and biallelic *MC4R* variants (36.4 ± 8.4 kcal per kilogram of lean mass) and patients with biallelic *LEPR* mutations (approximately 35 kcal per kilogram of lean mass) while the first value lies below this range [10, 11]. However, hyperphagia seems to be an age-dependent symptom which manifests more pronouncedly in younger children and decreases throughout adolescence [10]. A cohort of minors (< 18 years) with heterozygous loss-of-function mutations in *MC4R* consumed an average of 28.9 ± 2.9 kcal per kilogram of lean mass in an ad libitum test meal [6]. Patients of the same cohort who were under the age of 10 years consumed an average of 37.0 ± 3.2 kcal per kilogram of lean mass [6, 10]. A similar observation was made for patients with Prader-Willi-Syndrome: Some patients report a reduced preoccupation with food and some no longer experience hyperphagia at all in adulthood [20]. Therefore, it seems possible that hyperphagia might have been more pronounced in the first patient when he was younger. Alternatively, he might have learned coping strategies during previous lifestyle interventions and, while still experiencing hunger, is better able to control the drive to eat. Additionally, it should also be considered that the above-mentioned methods to assess hyperphagia do not sufficiently account for other components of eating behavior such as impulse control [2]. Currently, there is a lack of standardized tools for the comprehensive assessment of all aspects of eating behavior across all age groups. Therefore, the question whether the discrepancy between the two patients’ caloric intake in the test meal and the discrepancy between the older patient’s caloric intake and Dykens score are due to a regression of hyperphagia towards adulthood or due to the methods used to assess hyperphagia remains unanswered.

In the literature, early weight gain trajectories of biallelic non-syndromic forms of obesity demonstrate excessive and rapid weight gain in the first two years of life [1, 15, 30]. In the case of children with biallelic *LEP*, *LEPR* and *MC4R* variants, BMI trajectories in early childhood showed a particularly rapid increase, reaching BMI values of 25.8 kg/m^2^ (*LEP*), 25.4 kg/m^2^ (*LEPR*) and 22.8 kg/m^2^ (*MC4R*) by the age of six months [30]. Notably, patients with biallelic *POMC*, monoallelic non-syndromic and syndromic forms of obesity exhibit a slightly later onset of obesity and a more gradual weight gain compared to biallelic non-syndromic forms [1]. For instance, children with monoallelic *MC4R* variants or biallelic *POMC* variants reach BMI values of 22.8 kg/m^2^ and 19.6 kg/m^2^ by the age of six months respectively [30]. In the first patient, drastic weight gain started at the age of six years. This later age of onset has previously been observed in children with syndromic forms of obesity [1]. The second patient had a BMI of 20.1 kg/m^2^ at the age of five months which is in the range of children biallelic *POMC* and monoallelic *MC4R* variants. The difference in age of onset between the two cases may be explained by the fact that the second patient’s father, unlike the first patient’s father, was affected by extreme obesity as well as his mother, suggesting that the effect of rare PTVs in *APBA1* may be further aggravated by a familial multifactorial background disposition towards obesity. In fact, it has been observed in population studies that both increased maternal and increased paternal BMI are associated with an increased BMI in children compared to children of parents without increased BMI [18]. Similarly, lifestyle and an obesogenic environment have been reported to intensify the effect of loss-of-function variants in *MC4R *[25]. Additionally, incomplete penetrance may account for some of the variability in the phenotype in our patients. While penetrance of biallelic forms of monogenic obesity is assumed to be 100%, estimates on the penetrance of monoallelic forms gave varied values between 3 and 100% [8]. Furthermore, an age-dependent increase in penetrance was observed for *MC4R* variant carriers and thus a similar effect may also apply to *APBA1* variants [25].

Notably, the study examining the adults of the All of Us Research Program cohort also reported a link between *APBA1* loss-of-function variants and temporomandibular joint disorders [23]. In our two cases, we did not find any evidence for this disorder. Instead, a neurodevelopmental aspect appears to contribute to the phenotype in our patients. The first patient was diagnosed with impaired expressive language development, dyslexia and has a borderline average to slightly reduced IQ. This neurological phenotype, particularly the dyslexia, is shared by the patient's mother who is also carrier of the *APBA1* variant. The second patient displayed impaired expressive language development and was not able to form full sentences at the age of six years. In a previous genome-wide association study conducted on the UK Biobank cohort, *APBA1* was found to have a significant association to cognitive functions [7]. Therefore, it seems possible that a deficiency in this gene might cause neurological deficits such as dyslexia or impaired speech development. At present time, this hypothesis and the role of *APBA1* in the regulation of neurodevelopment, weight, hunger and satiety need to be proven and investigated through further experiments, respectively.

This study is further limited by the fact that only two patients could be identified and phenotyped. Due to the very small sample size it is difficult to reliably identify a recurring phenotype associated with PTVs in *APBA1* in childhood. In particular in the second case, the child’s father was neither available for genetic testing nor for clinical assessment. The mother reports that he had severe obesity and underwent bariatric surgery. Therefore, we cannot rule out a monogenic cause of obesity in him that may contribute to the son’s phenotype. However, we have not found any likely pathogenic or pathogenic variants in known obesity genes in the son that would indicate he inherited a known causative variant from the father. Future investigations into the effect of PTVs in *APBA1* should therefore include both clinical and experimental approaches. The evaluation of larger cohorts of children and adolescents with severe obesity may for example identify further cases with rare PTVs in *APBA1* and provide the basis for a more detailed phenotypic characterisation. Furthermore, functional in vitro studies could illuminate the molecular mechanism that connects *APBA1* to the regulation of hunger and satiety. In vitro studies could also provide a way to asses the effect of individual genetic variants on splicing and protein function.

## Conclusion

In summary, we identified rare heterozygous potentially protein-truncating variants in *APBA1* in two minors as possible cause of severe childhood obesity. This phenotypic finding stands in contrast to the previous report from Zhao et al. that postulated that genetic variants in *APBA1* are only associated with adult-onset obesity [27]. Furthermore, phenotyping of the two affected minors revealed evidence of hyperphagia, suggesting an altered regulation of hunger and satiety as well as neurodevelopmental features, such as dyslexia or impaired speech development. Despite the fact that the role of *APBA1* in the regulation of weight remains unclear, and the fact that penetrance of obesity in childhood might be incomplete, we advocate that rare PTVs in *APBA1* are associated with a genetic form of obesity not only in adults but also in children. Thus, *APBA1* should not be ignored in the diagnostic process for monogenic obesity in childhood.

## Data Availability

Source data and unique materials used in this study are available from the corresponding author upon reasonable request.

## Abbreviations

APBA1: Amyloid beta A4 precursor protein-binding family A member 1
BSN: Bassoon
BMI: Body mass index
EEG: Electroencephalogram
HbA1c: Hemoglobin A1C
IGV: Integrative Genomics Viewer
IQ: Intelligence quotient
KCAL: Kilocalories
LEP: Leptin
LEPR: Leptin receptor
MC4R: Melanocortin 4 receptor
NGS: Next-generation sequencing
NMDA: N-methyl-D-aspartate
POMC: Proopiomelanocortin
PTV: Protein-truncating variant
SDS: Standard deviation score
TSH: Thyroid-stimulating hormone
